# Professor Eve’s Card

**Published:** 1850-12

**Authors:** 


### PROFESSOR EVE'S CARD.

In view of Professor Eve's change of residence, from Augusta, Ga., to Louisville, Ky., and his expected return to Georgia at the close of the present lecture term, he requests the insertion of the following card. Editors of Medical Journals will confer a favor by inserting Professor Eve's card, in their respective periodicals.

*American Surgery*, as well as *Practical Medicine*, is in good hands, and we hope the surgeons of the Union will do all in their power to aid Professor Eve in presenting a report creditable to the profession, to the American Medical Association, and the high reputation of the distinguished chairman. B.

"*Surgical Report for the American Medical Association.*—The committee is invited to meet in the Charleston Hotel, South Carolina, the evening of the first Tuesday in May next. All professional brethren, who have *surgical* facts connected with the improvement of this branch of the profession during the year, will please address them to the chairman of the committee by the first of April, at *Augusta, Georgia.* As all cannot be reached by a circular, it is hoped no one will wait for a more direct application than this general invitation.

"By extending this notice, the medical periodicals of our country will advance the interests of the American Medical Association, and the editors will confer a favor upon their recent confrêre,

"PAUL F. EVE, M.D.,

"Prof. of Surgery in the Louisville University, and Chairman of the American Medical Association.

"LOUISVILLE, Ky., Dec. 1850."

